# Near-Complete Genome Sequence of Avian Influenza Virus Strain A/mallard/Balkhash/6304/2014 (H1N1) from Kazakhstan

**DOI:** 10.1128/MRA.00248-19

**Published:** 2019-05-02

**Authors:** Kobey Karamendin, Aidyn Kydyrmanov, Saule Asanova, Elizaveta Khan, Klara Daulbayeva, Yermukhammet Kasymbekov, Marat Sayatov

**Affiliations:** a Laboratory of Viral Ecology, Institute of Microbiology and Virology, Almaty, Kazakhstan; DOE Joint Genome Institute

## Abstract

An avian influenza virus strain, A/mallard/Balkhash/6304/2014 (H1N1), was isolated during a wild bird monitoring study in Kazakhstan in 2014. The virus was isolated from a wild mallard duck (Anas platyrhynchos) in eastern Kazakhstan. Here, we present the near-complete genome sequence of the virus.

## Abstract (precis)

An avian influenza virus strain, A/mallard/Balkhash/6304/2014 (H1N1), was isolated during a wild bird monitoring study in Kazakhstan in 2014. The virus was isolated from a wild mallard duck (Anas platyrhynchos) in eastern Kazakhstan.

## ANNOUNCEMENT

Avian influenza virus (AIV) is a single-stranded segmented negative-sense RNA virus belonging to the *Orthomyxoviridae* family, genus Influenzavirus A (1). Continuous circulation of high- and low-pathogenic AIV in wild birds and their subsequent spread around the world have raised a question on the role of wild birds as a global transmission factor (2). H1N1 is an AIV subtype that circulates among wild birds but has the potential for adaptation to humans and other mammals (3).

The A/mallard/Balkhash/6304/2014 (H1N1) virus strain was isolated from a cloacal swab sample from a wild mallard duck after inoculation into 10-day-old embryonated chicken eggs. Allantoic fluid was checked for the presence of hemagglutinating agents by using a hemagglutination assay with chicken red blood cells (4). Viral RNA was extracted using the QIAamp viral RNA minikit (Qiagen). Library preparation was conducted using the NEBNext Ultra RNA kit along with the rRNA depletion kit (NEB, USA), according to the manufacturer’s instructions. Paired-end sequencing of multiple pooled samples was performed on a MiSeq instrument using the MiSeq reagent kit v3 (Illumina). Sequencing quality analysis was performed with FastQC (5). In total, approximately 1,571,000 raw sequencing reads were obtained, with a GC content of 46.1%. Sequence data were trimmed, assembled, and mapped against the reference sequences downloaded from GenBank using the Geneious 11.0 software (Biomatters), with default parameters. The AIV reference sequences with the highest genome coverage were selected for analysis. The final assembly, obtained with Geneious 11.0 (default parameters), was 13,160 nucleotides in length, with a mean coverage of 17,320-fold. The size of each segment of the virus is shown in Table 1. The obtained sequences were aligned using the MEGA 7.0 software (6). Phylogenetic trees were constructed using the neighbor-joining method and the Tamura-Nei model (7), also in the MEGA 7.0 software.

**TABLE 1:** Genome characteristics of isolate A/mallard/Balkhash/6304/2014 (H1N1)

Gene/segment	Size (nucleotides)	GC content (%)	Strain with closest relative sequence	Identity at nucleotide level (%)	GenBank accession no.
PB2	2,280	46.01	A/duck/Mongolia/154/2015 (H1N2)	98.07	LC121273
PB1	2,253	43.85	A/Anseriformes/Anhui/L25/2014 (H1N1)	97.82	KU881702
PA	2,160	44.40	A/duck/Bangladesh/24705/2015 (H7N1)	99.17	KY635807
HA	1,752	42.64	A/mallard duck/Georgia/11/2011 (H1N1)	98.34	MF146715
NP	1,520	47.30	A/mallard/Czech Republic/15902-17K/2009 (H6N2)	98.16	HQ244431
NA	1,358	44.62	A/duck/Moscow/4970/2013 (H1N1)	99.63	MF969259
M	965	49.95	A/duck/Mongolia/118/2015 (H4N6)	99.90	LC121263
NS	872	44.84	A/duck/Mongolia/655/2015 (H2N3)	99.20	LC121432

This study documents the isolation of AIV during a global spread of postpandemic swine H1N1 influenza virus, and it was interesting to compare a local avian strain with human pandemic strains. A BLAST search of hemagglutinin and neuraminidase gene sequences revealed their similarity at the nucleotide level to those of AIV strains isolated in Caucasus and Russia (Table 1). The deduced amino acid motif for the fusion cleavage site of the HA gene segment was PSIQSR, which corresponds to low pathogenicity (8). The Kazakh isolate contained avian-type receptor binding signatures E190 and G225 at HA but has the mammalian-type signature Q at position 226 that is connected to increased pathogenicity and transmissibility of influenza viruses in ferrets (9). It was suggested that these substitutions could enhance the ability of avian influenza viruses to adapt to mammalian hosts (10).

**FIG 1:**
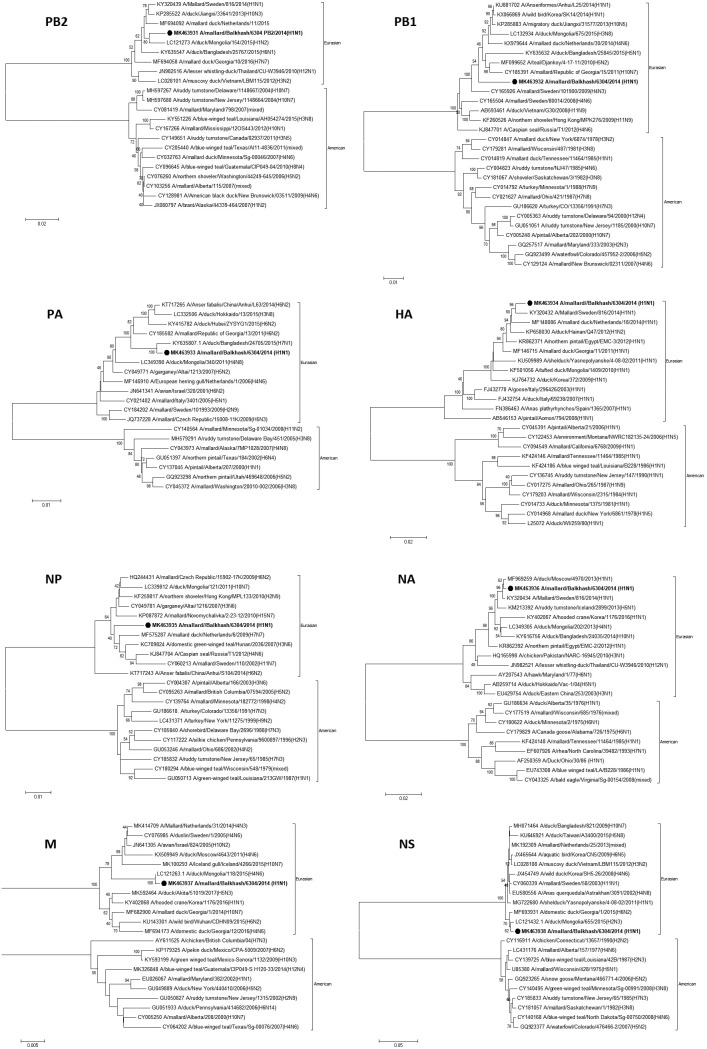
Phylogenetic trees for all genes of the isolate A/mallard/Balkhash/6304/2014 (H1N1).

The internal genes at the nucleotide level were 98 to 99% similar to those of viral strains isolated in Mongolia, the Czech Republic, China, and Bangladesh (Table 1). Phylogenetic analysis revealed that all gene segments of A/mallard/Balkhash/6304/2014 (H1N1) clustered with the Eurasian lineage AIVs of avian origin (Fig. 1).

### Data availability.

The complete genome sequence of A/mallard/Balkhash/6304/2014 (H1N1) is available at GenBank under the accession numbers MK463931 to MK463938. Raw sequence reads are available under BioProject accession number PRJNA525314.
